# Vaccination with Deglycosylated Modified Hemagglutinin Broadly Protects against Influenza Virus Infection in Mice and Ferrets

**DOI:** 10.3390/vaccines10081304

**Published:** 2022-08-11

**Authors:** Limin Zhang, Junyu Chen, Chenguang Shen, Guosong Wang, Zhen Lu, Dian Zeng, Ying Gao, Huiqing Chen, Ningshao Xia, Yixin Chen

**Affiliations:** 1 State Key Laboratory of Molecular Vaccinology and Molecular Diagnostics, National Institute of Diagnostics and Vaccine Development in Infectious Diseases, School of Life Sciences, Xiamen University, Xiamen 361102, China; 2 State Key Laboratory of Molecular Vaccinology and Molecular Diagnostics, National Institute of Diagnostics and Vaccine Development in Infectious Diseases, School of Public Health, Xiamen University, Xiamen 361102, China; 3 Guangdong Provincial Key Laboratory of Tropical Disease Research, School of Public Health, Southern Medical University, Guangzhou 510515, China

**Keywords:** universal influenza vaccine, Hemagglutinin, glycosylation, deglycosylation modification

## Abstract

Recent efforts have been directed toward the development of universal influenza vaccines inducing broadly neutralizing antibodies to conserved antigenic supersites of Hemagglutinin (HA). Although several studies raise the importance of glycosylation in HA antigen design, whether this theory can be widely confirmed remains unclear; which influenza HA with an altered glycosylation profile could impact the amplitude and focus of the host immune response. Here, we evaluated the characteristics and efficacy of deglycosylated modified HA proteins, including monoglycosylated HA (HA_mg_), unglycosylated HA (HA_ug_), and fully glycosylated HA (HA_fg_), without treatment with H3N2 Wisconsin/67/2005. Our results showed that HA_ug_ could induce a cross-strain protective immune response in mice against both H3N2 and H7N9 subtypes with better antibody-dependent cellular cytotoxicity (ADCC) than the HA_mg_- and HA_fg_-immunized groups, which suggested that highly conserved epitopes that were masked by surface glycosylation may be exposed and thus promote the induction of broad antibodies that recognize the hidden epitopes. This strategy may also supplement the direction of deglycosylated modified HA for universal influenza vaccines.

## 1. Introduction

The influenza virus is a rapidly evolving pathogen that remains a serious threat to public health worldwide despite the availability of licensed vaccines. Influenza vaccination is one of the most effective means of preventing influenza infection and disease. Currently, influenza vaccines are composed of H1N1 and H3N2 of influenza A virus and either lineage (Trivalent influenza vaccine, TIV) or both lineages (Quadrivalent influenza vaccine, QIV) of influenza B virus, which were recommended by the World Health Organization (WHO). The low protective effect of the current influenza vaccines was mainly due to the significant mismatch between circulating and recommended strains, especially against H3N2 viruses with rapid antigenic drifts [1,2,3]. Thus, it is necessary to develop an efficient vaccine that provides broad-spectrum protection against a variety of influenza viruses.

Hemagglutinin (HA) protein mediates the membrane fusion of viral and host cells and thus is regarded as the primary vaccine target [4,5,6,7]. However, the antibodies induced by traditional influenza vaccines usually cannot effectively neutralize subtypes or emerging strains due to the rapid mutation in HA through antigenic drift and reassortment [8]. Thus, the vaccine must be updated annually. Recent efforts have been directed toward the development of universal influenza vaccines with broadly neutralizing responses to conserved antigenic supersites of HA [9].

The immunodominant response elicited against influenza HA often targets variable epitopes on the head domain of HA, which is ready for escape mutation [10]. Thus, the primary challenge is to direct the immune response away from antigenically variable epitopes to subdominant but less variable and more broadly protective epitopes. Strategies including HA-stem nanoparticle vaccine [11], the trimeric HA stem vaccine [12], and chimeric HAs with head domains from different strains for sequential vaccination [13] mainly focus on rational immunogen design aimed at altering patterns of immunodominance. A computational design approach has been used to selectively present conserved epitopes by engineering immunogens with scaffold displays [14]. In short, the mainstream strategy is to entirely remove the immunodominant epitopes from the native glycoproteins to focus the immune response on subdominant and broadly protective epitopes.

Glycosylation is a common modification that plays an important role in protein biology [15]. Since the introduction or removal of glycans in the HA protein has caused the virus to evolve to escape from the host immune response, several studies have focused more attention on how glycosylation affects immunodominance in HA antigen design [16,17,18,19]. For example, a glycan shield approach by introducing a hyperglycosylated mutation can elicit serum responses with limited breadth and neutralization [18]. Furthermore, Wong et al. reported that a monoglycosylated H1N1 HA-based vaccine (HA_mg_) trimmed by glycosidase could elicit a cross-strain immune response [20]. This study opens the door to the possibility of designing an engineered HA glycoprotein to expose protective but occluded epitopes and change the immune response with glycan modification. Here, to explore whether this glycan modification strategy can be applied in group two of influenza A virus, we evaluated the efficacy of deglycosylated modified HA proteins from H3N2 with various lengths of glycans as potential vaccine candidates. Interestingly, immunization of the deglycosylated modified HA in mice was found to elicit higher levels of antibody-dependent cellular cytotoxicity (ADCC) activities and stronger T-cell responses and conferred broad spectrum protection against homologous and heterosubtypic strains of H3N2 and H7N9, especially the unglycosylated form of HA (HA_ug_) rather than HA_mg_. Similar results could be observed in ferret models.

## 2. Materials and Methods

### 2.1. Cells and Virus

Madin-Darby Canine Kidney (MDCK) cells were obtained from the American Type Culture Collection (ATCC). MDCK cells were maintained in Dulbecco’s modified Eagle’s medium (DMEM)-high glucose (Sigma Aldrich, St. Louis, MO, USA) supplemented with 10% low endotoxin fetal bovine serum (FBS) (Cegrogen Biotech, Stadtallendorf, Germany). The influenza virus strains A/Aichi/1968 (H3N2), A/Beijing/32/1992 (H3N2), and Wisconsin/67/2005 were kindly provided by BEI Resources. The influenza virus A/Shanghai/02/2013(H7N9) × A/PR/8/34 reassortant was obtained from the University of Hong Kong. The mouse-adapted strains A/Aichi/1968(H3N2), A/Beijing/32/1992(H3N2), and A/Shanghai/02/2013(H7N9) (HA, NA) × A/PR/8/34 reassortant were generated in our laboratory through successive passaging in the lungs of mice. A/Aichi/1968(H3N2) virus was serial passaging (up to 7 passages) through the lungs of BALB/c mice, and the MLD_50_ (50% mouse lethal dose) is 1 × 10^5^ TCID_50_; A/Beijing/32/1992(H3N2) virus was serial passaging up to 14 passages, and the MLD_50_ is 5 × 10^4^ TCID_50_; A/Shanghai/02/2013(H7N9) (HA, NA) × A/Puerto Rico/8/1934 (H3N2) reassortant was serial passaging up to 8 passages, and the MLD_50_ is 5 × 10^5^ TCID_50_. All viruses were grown in MDCK cells using standard viral culturing techniques.

### 2.2. Construction, Expression and Purification of HA Protein

The H3 HA sequences were downloaded from the GISAID database. The transmembrane domain was replaced with the residues that are at the bacteriophage T4 fibritin trimerization segment, the thrombin cleavage site, and the His-tag at the C-terminus of HA. The nucleotide sequence of HA from A/Wisconsin/67/2005 (H3N2) was synthesized with codons optimized by Sangon (Shanghai, China). The gene was inserted into the *Kpn*I and *Xba*I restriction sites of the pCMV vector for human cell expression. HA protein was expressed and purified from 293F cells. All HA proteins were cloned, expressed, and purified as above.

Potential N-linked glycans were predicted using the NetNGlyc server. HA_mg_ was obtained by treatment with Endo H from New England Biolabs of the full-length HA, which was incubated with 200 Units of Endo H/mg protein at 37 °C for 16–18 h. HA_ug_ was obtained by treatment with PNGase F (NEB, Ipswich, MA, USA), which added 10 × G7 reaction buffer, 10% (*v*/*v*) Nonidet P-40, and 500 units of PNGase F at 37 °C for 16–18 h.

### 2.3. Vaccinations and Challenges

Female 6- to 8-week-old BALB/c mice (5 per group) were immunized twice with 15 μg HA_fg_, HA_mg_ and HA_ug_ proteins in 50 μL Tris buffer (20 mM Tris base, pH 7.4) mixed with 50 μL aluminum adjuvant at days 0 and 14. Serum samples were collected at day 28 for serological analysis. At day 42, immunized mice were challenged with 25 MLD_50_ of the homologous viruses A/Beijing/32/1992(H3N2) and A/Aichi/1968(H3N2) obtained from BEI Resources and heterologous virus A/Shanghai/02/2013(H7N9) × A/PR/8/34 reassortant obtained from the University of Hong Kong intranasally. PBS-immunized mice were used as a mock control. Mice were sacrificed at 3 dpi and 6 dpi to determine lung virus titers, which were determined by TCID_50_ assay. Survival rates and body weights were monitored daily for 14 days. Twenty-five percent weight loss was used as a humane endpoint for challenge. Any mice reaching that endpoint were euthanized.

Groups of four female 16- to 18-week-old ferrets were immunized twice in intramuscular route with 30 μg HA_fg_, HA_mg_ and HA_ug_ proteins in 300 μL Tris buffer mixed with 300 μL aluminum adjuvant on days 0 and 28. Serum samples were collected at day 42 for serological analysis. On day 56, immunized ferrets were challenged with 50 LD_50_ of the homologous viruses A/Beijing/32/1992(H3N2) intranasally. Veterinary clinical symptoms were observed every day. Nasal washes were collected every other day for 7 days. Virus titrations were obtained from nasal washes titrated by TCID_50_.

### 2.4. Serological Analysis

Serum samples were collected 28 days after the booster immunization and treated with receptor destroying enzyme (RDE) (Denka Seiken, Japan) at 37 °C for 18 h followed by inactivation of the RDE via incubation at 56 °C for 30 min for serological analysis.

ELISA was performed using standard ELISA procedures. Dilutions of immunized sera were incubated for 45 min at 37 °C on 96-well plates coated with 10 ng inactivated virus by ultracentrifugation. Binding was visualized by incubation with HRP-conjugated anti-IgG, -IgG1, or -IgG2a isotype antibodies (Southern Biotech, Birmingham, AL, USA) for 45 min at 37 °C. BM Chemiluminescence ELISA Substrate (Roche Applied Sciences, Indianapolis, IN, USA) was added to the plates. Plates were then read on a Lmax plate reader (Molecular Devices, Sunnyvale, CA, USA). Between each step, plates were washed with PBST.

For the hemagglutination inhibition assay, RDE-treated sera were serially diluted 2-fold in 96-well plates and incubated with 8 HA units of virus for 30 min at room temperature. HAI titers were measured by the addition of 50 µL of 0.5% turkey red blood cells or 0.75% guinea pig erythrocytes by incubation for 45 min at room temperature.

For the microneutralization assay, RDE-treated sera were mixed with 200 TCID50 of virus and were then incubated for 1 h at 37 °C. The mixture was added to confluent MDCK cells in MEM supplemented with 2 μg/mL 6-(1-tosylamido-2-phenyl) ethyl chloromethyl ketone (TPCK)-treated trypsin in a 96-well plate and incubated at 37 °C for 48 h. HA titers were used to determine the neutralization titers by HA assay.

### 2.5. ADCC and Complement Dependent Cytotoxicity (CDC) Assay

MDCK cells infected with virus at a multiplicity of infection (MOI) of 10 were used as target cells. The target cells were plated at a concentration of 3 × 10^4^ cells per well in 96-well plates (in duplicate) and incubated for 12 h at 37 °C with 5% CO_2_. The cells were then washed twice with PBS and replenished with 100 μL per well of assay media (DMEM).

For the ADCC assay, the target cells labeled with PKH67 (Sigma-Aldrich) were washed twice with PBS and plated in triplicate at 5 × 104 cells per well in 96-well plates, followed by the addition of serially diluted RDE-treated sera. After incubation for 30 min at 37 °C, human PBMCs were added to the target cell-antibody mixture at 1 × 106 cells per well. Following 3 h of incubation at 37 °C, 1 μL of 7-AAD (eBioscience) was added to the wells to stain dead cells. Cell death was determined on a FACS Aria III flow cytometer using BD FACS Diva software (BD Biosciences). For the CDC assay, serially diluted RDE-treated sera were added to each well, followed by 50 μL of human serum complement diluted in PBS (Quidel), and the cells were incubated for 2 h. Finally, 10 μL of cell-counting kit reagent (CCK-8, Dojindo) was added to each well, and the plates were incubated for 48 h at 37 °C and 5% CO_2_. The absorbance was read on a microplate reader to determine cell viability based on dye-reducing activity levels. In order to calculate the percent of CDC, the following calculation was used: cytotoxicity (%) = (OD of baseline reaction − OD of experimental reaction)/(OD of baseline reaction − OD of maximal reaction) × 100. Target cells treated with complement alone were used to determine the baseline reaction value. Target cells treated with complement and sera were used to determine the experimental reaction value, and target cells lysed in 1% Triton X-100 were used to determine the maximal reaction value2.6. Flow cytometry

The ratios of influenza virus-specific IFN-γ-secreting cells in CD8+/CD4+, and IL-4 secreting cells in CD4+ T-cell levels were determined by flow cytometry. Mouse spleens collected on day 28 after prime-boost immunization were dissected and passed through a 40 mm cell strainer (BD Biosciences, San Jose, CA, USA). The splenocytes were seeded at 1 × 10^6^ cells/well and stimulated with 5 µg/mL HA, and anti-mouse CD28 (10 µL/mL) was added for co-stimulation. After 5 h of incubation with antigens (37 °C and 5% CO_2_), the cells were washed with PBS, and the remaining erythrocytes were lysed with a commercially available hemolysis buffer (Morphisto, Frankfurt am Main, Germany).

Isolated splenocytes were washed with FACS staining buffer, stained for 30 min with fluorescein isothiocyanate (FITC)-labeled rat anti-mouse monoclonal antibodies (BD Biosciences) against the CD4 and CD8 cell surface markers, fixed and permeabilized with Cytoperm/Cytofx solution. Later, the cells were washed and treated with phycoerythrin-conjugated rat anti-mouse IFN-γ antibody for 30 min at RT in the dark. Analysis was performed on a FACS Calibur flow cytometer (BD Biosciences).

### 2.6. Statistical Analyses

Statistical significance was assigned when *p* values were <0.05 using GraphPad Prism 8.0 (GraphPad Software, Inc., San Diego, CA, USA). The significance of differences in neutralization/antibody titers and mean fractional body weights of surviving mice between different groups were analyzed by analysis of variance and Student’s *t* test. Statistical significance in all figures is expressed as *, *p* < 0.05; **, *p* < 0.01; and ***, *p* < 0.001, **** *p* < 0.0001.

## 3. Results

### 3.1. Deglycosylated Modified HA Proteins Treated with Different Glycosidases Could Retain Structural Integrity and Primary Epitope Conformations

To analyze the potential sites for glycan modification, we found that the number of glycosylation modifications of H3N2 subtype influenza A viruses increased from 7 to 11 during their evolution in humans from 1968 to the present (Figure S1A,B). While antigenic variation, including glycan introduction or removal, is among the mechanisms viruses have evolved to escape host immunity [21,22], we used H3N2 Wisconsin/67/2005 HA with 11 putative N-linked glycosylation sites (PNGs) as a template for further analysis. HA glycosylation sites were predicted, including 79 NST, 142 NWT, 149 NGT, 160 NNS, 181 NVT, and 262 NST in the globular domain, as well as 24 NST, 38 NGT, 54 NAT, 301 NGS, and 499 NGT in the stalk domain. Interestingly, the PNGs overlapped with the antigenic and conserved sites on the HA protein surface, regardless of whether they were located at the head domain or stem domain (Figure S1C).

**Figure 1:**
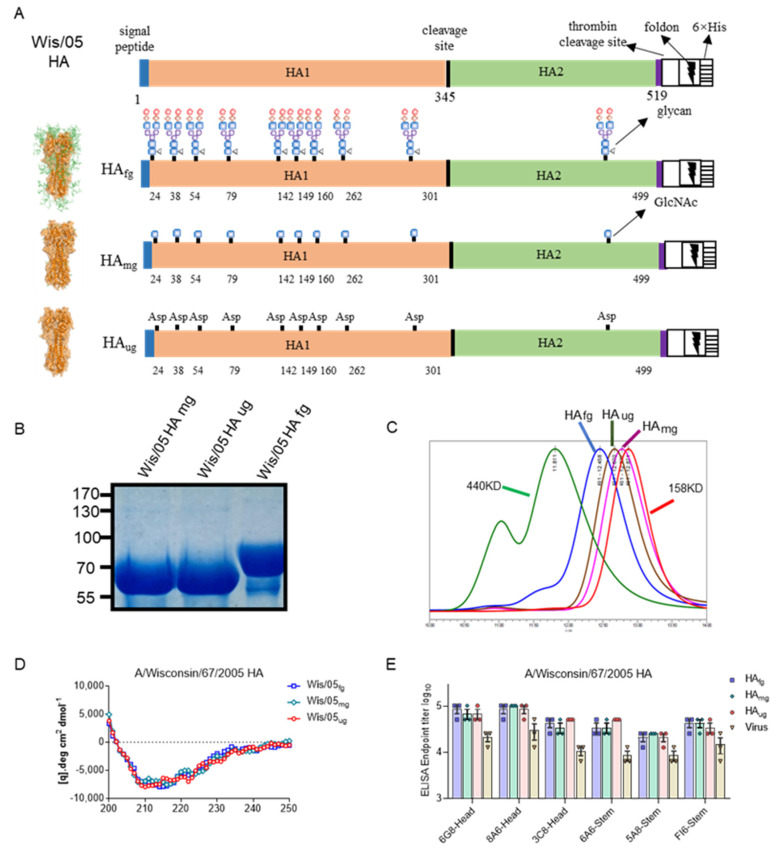
Deglycosylated modified HA proteins treated with different glycosidases could retain structural integrity and primary epitope conformations. (**A**) Schematic representation of the Wis/05 (H3N2) HA proteins with different lengths of glycans at the 11 glycosylation sites: HA_fg_ with the typical complex type N-glycans; HA_mg_ was obtained by treatment with Endo H, only a GlcNAc at its N-glycosylation sites; HA_ug_ was obtained by treatment with glycosidase PNGase F without glycans at its N-glycosylation sites. Schematic of the wild-type and glycosidase-modified HAs modeled using PyMOL. (**B**) SDS-PAGE analysis of wild-type and glycosidase-treated HAs. When HAs were deglycosylated with Endo H and PNGase F, the molecular weight of HA was expected to be decreased. (**C**) SEC analysis of glycosidase-treated HA showed that deglycosylated HAs still maintained a trimeric state. (**D**) Circular dichroism (CD) structural analysis indicated that glycosidase-treated HA retained its structural integrity. (**E**) Conformational integrity analysis indicated no significant difference in the affinity of glycosidase-treated HA to both HA head- and stalk-specific antibodies. Several representative antibodies targeting the head or stem domain of HA proteins produced in our lab were used for antigenic assessment of major epitopes.

In this study, we prepared native fully glycosylated HA (HA_fg_) from A/Wisconsin/67/2005 (H3N2) expressed by human embryonic kidney cells (HEK293). Monoglycosylated HA (HA_mg_) is obtained after treatment with the glycosidase Endoglycosidase H (Endo H) with GlcNAc only, and unglycosylated HA (HA_ug_) is obtained after treatment with the glycosidase PNGase F, which is denoted deglycosylated forms with amino acid change from asparagine (Asn) to aspartic acid (Asp) in each glycosylation site (Figure 1A). To ensure purity and glycan composition after separate filtration and concentration, the deglycosylated modified HAs proved to have decreased molecular weight size compared to HA_fg_ with trimeric forms (Figure 1B,C). The secondary structures of HA_mg_ and HA_ug_ show few differences from HA_fg__,_ as judged by circular dichroism measurements (Figure 1D). In addition, the antigenicity assay showed that HA_fg_ and deglycosylated modified HAs could be well recognized by representative antibodies targeting both the head domain or stem domain produced in our lab, which indicated that the major epitopes remained unchanged between the fully glycosylated HA and deglycosylated modified HAs (Figure 1E). In order to test antiviral efficacy and the protective capacity of the immune responses induced by deglycosylated modified HAs as vaccine candidates, two different animal models were used.

### 3.2. Vaccination of Mice with Deglycosylated Modified HA Elicits a Strong T-Cell Response and Antibody-Dependent Effector Functions against H3 and H7 Viruses

**Figure 2:**
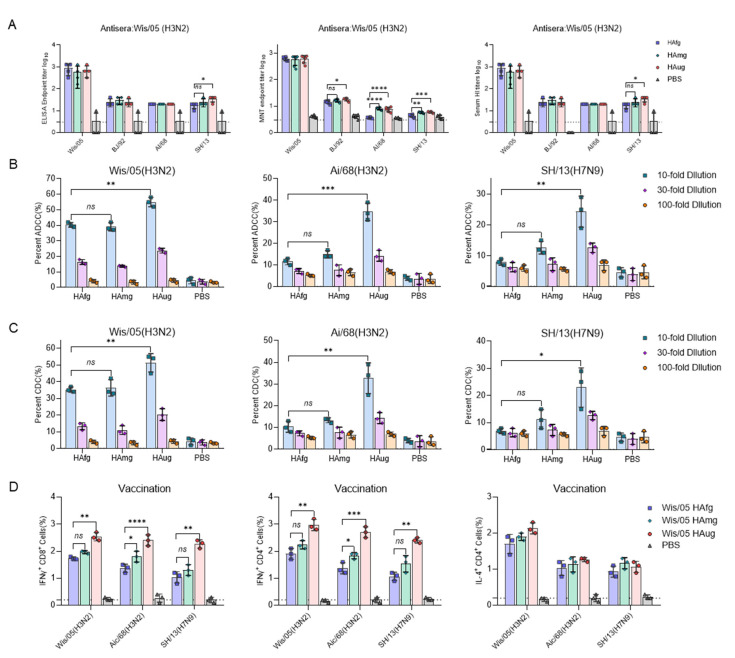
Vaccination of mice with deglycosylated modified HA elicits a strong T-cell response and antibody-dependent effector functions against H3 and H7 viruses. BALB/c mice (six mice in each group) were immunized with 15 µg of HA_fg_, HA_mg_ and HA_ug_ on days 0 and 14. The virus-specific B-cell and T-cell responses were measured on day 28 after boost immunization. (**A**) ELISA endpoint titer, hemagglutination inhibition (HAI) titers and neutralizing antibody titers of immune sera were determined against Wis/05 (H3N2), Vic/11 (H3N2), BJ/92 (H3N2), AI/68 (H3N2), and SH/13 (H7N9). (**B**) ADCC activity of immune sera against Wis/05(H3N2), AI/68(H3N2) and SH/13(H7N9) virus target MDCK cells was measured by flow cytometry using a rapid fluorometric ADCC assay. (**C**) The virus-specific complement-dependent cytotoxicity (CDC) activity of the sera from immunized mice was determined by a complement-mediated cell killing assay. (**D**) Single splenocytes from immunized mice were harvested for the analysis of IFN-γ- and IL-4-secreting T cells determined by an enzyme-linked immunospot (ELISPOT) assay stimulated by specific HA proteins from Wis/05(H3N2), AI/68(H3N2) and SH/13(H7N9). Data are presented as the mean ± SEM. The results were calculated with Prism software using Student’s *t* test; significant differences are marked as * *p* < 0.05; ** *p* < 0.01; *** *p* < 0.001; **** *p* < 0.0001.

We next sought to evaluate the impact of glycosidase modification on the immunogenicity of HA in vivo. To detect whether more broadly neutralizing activity was induced by the vaccine, we performed ELISA binding assays, HAI assays, and neutralizing assays against A/Wisconsin/67/2005 (Wis/05), A/Beijing/32/1992 (BJ/92), A/Aichi/1968 (AI/68), and A/Shanghai/02/2013 (SH/13) (Figure 2A). Sera of mice were collected at 28 days postinoculation for evaluation of the humoral immune response. Unexpectedly, the antiserum from HA_ug_ showed slightly better binding and neutralizing activity to SH/13, and no significant difference was observed between HA_fg_ and HA_mg_ with low levels of antibody titers. These data indicate that the deglycosylated modified HA could not elicit cross-neutralizing antibodies against H3N2 and H7N9. In addition to antibody-mediated neutralization, Fc-mediated effector functions also play an important role in protection against influenza infection [23,24]. Upregulation of IgG2a and the balance of Th1/Th2 ratio is often correlated with enhancement of ADCC function.HA_ug_ group induced a lower IgG1/IgG2a ratio than the HA_fg_ group of antiserum against SH/13(H7N9), indicating higher Th1-biased antibody responses to the heterotypic virus, while a higher IgG1/IgG2a ratio was induced by HA_ug_ group of antiserum against homotypic virus BJ/92(H3N2) strain (Figure S2). Sera from vaccinated mice were assessed for the presence of the capacity of antibodies elicited by vaccination to participate in ADCC and CDC effector functions (Figure 2B,C). Interestingly, only HA_ug_-vaccinated mice could induce comparable levels of ADCC activity against Wis/05, AI/68, and SH/13 virus, whereas HA_fg_ and HA_mg_ induced little ADCC activity. Similar results were also observed in CDC assays against Wis/05, AI/68, and SH/13 viruses.

To further evaluate the role of antigen-specific cytokine-secreting cells in immunized mice, the splenocytes were collected on day 28 after prime-boost immunization, and the expression of influenza-virus-specific IFN-γ+CD8+, IFN-γ+CD4+, and IL-4+CD4+ T cells were detected with specific HA proteins from Wis/05, AI/68, and SH/13 for stimulation in all three vaccinated groups. As shown in Figure 2D, the three vaccinated groups produced similar levels of IL-4+CD4+ secreting cells. However, more IFN-γ+CD8+ and IFN-γ+CD4+ secreting cells were elicited in the HA_ug_-vaccinated group (Figure 2D). These results confirmed that HA_ug_ could induce more robust FC-dependent functional antibodies by stimulating more CD4 and CD8 T-cell responses than HA_mg_ and HA_fg_.

### 3.3. Deglycosylated Modified HAs as Vaccine Candidates Have Broad-Spectrum Antiviral Activity against Homotypic and Heterosubtypic Influenza A Virus in Mice

**Figure 3:**
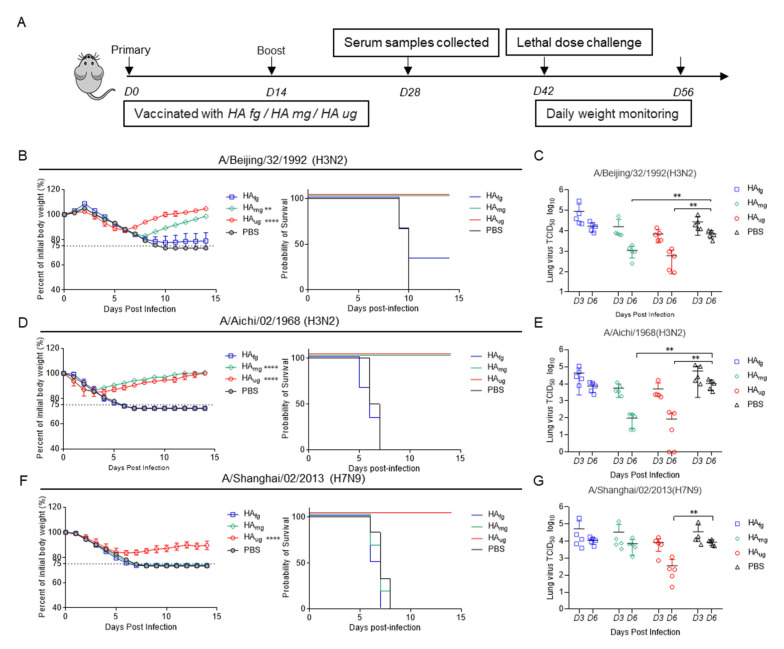
Deglycosylated modified Has as vaccine candidates have broad-spectrum antiviral activity against homotypic and heterosubtypic influenza A virus in mice. (**A**) Timeline of vaccination and challenge experiments in mice. Six- to eight-week-old female BALB/c mice (six mice in each group) were intramuscularly injected with 15 µg of HA_fg_, HA_mg_ and HA_ug_ on days 0 and 14 and challenged on day 42 intranasally with 25 MLD_50_ (50% mouse lethal dose) of A/Beijing/32/1992(H3N2) virus (**B**), A/Aichi/02/1968(H3N2) virus (**D**), and A/Shanghai/02/2013(H7N9) virus (**F**). Mice were monitored daily for weight loss change in each group over 14 days post-infection. Mice were euthanized when they lost 25% of their original body weight. (**C**,**E**,**G**) Virus titers were determined for lung tissues by TCID_50_ assay at 3 dpi and 6 dpi post infection. Data are presented as the mean ± SEM. The results were calculated with Prism software using Student’s *t* test and two-way ANOVA; significant differences are marked as ** *p* < 0.01; **** *p* < 0.0001.

In order to verify the antiviral effect of deglycosylated HA proteins, six-week-old female BALB/c mice were intramuscularly immunized twice as indicated with a two-week interval with 15 μg HA_fg_, HA_mg_, and HA_ug_ (Figure 3A) and challenged by intranasal inoculation with lethal doses with 25 MLD_50_ (50% mouse lethal dose) mouse-adapted viruses of A/Beijing/32/1992(H3N2), A/Aichi/1968(H3N2) and A/Shanghai/02/2013(H7N9). Mice vaccinated with PBS were used as controls. The efficacy of vaccine protection was evaluated for 14 days by recording the survival rate and body weight change (Figure 3B,D,F). For the BJ/92 challenge group, the control mice succumbed to infection after ten days, and only 33% (2/6) of the HAfg group survived, while both the HA_ug_ and HA_mg_ groups showed full protection. For the antigenically mismatched H3N2 AI/68 challenge group, all the mice in the HA_fg_ group succumbed to infection after seven days compared to the control group, and both the HA_ug_ and HA_mg_ groups still provided complete protection with mild weight loss. Remarkably, only mice vaccinated with HA_ug_ were completely protected (100% survival) against heterosubtypic group two H7N9 SH/13 virus infection, in contrast to the 0% survival of HA_fg_, HA_mg,_ and control groups.

Another group of mice was assessed for virus clearance conferred by vaccinated mice at 3 and 6 dpi (Figure 3C,E,G). The HA_mg_- and HA_ug_-immunized mice had significantly reduced lung virus titers at 6 dpi in the case of the H3N2 virus challenge (Figure 3C). As expected, the viral load in the lung tissue of the HA_ug_-vaccinated mice significantly decreased compared to that in the HA_fg_ and HA_mg_ groups at 6 dpi (Figure 3G), which was consistent with the above survival rate data.

### 3.4. Glycosidase-Treated HA Vaccines Induce Protective Immunity against Influenza Virus Infection in Ferrets

**Figure 4:**
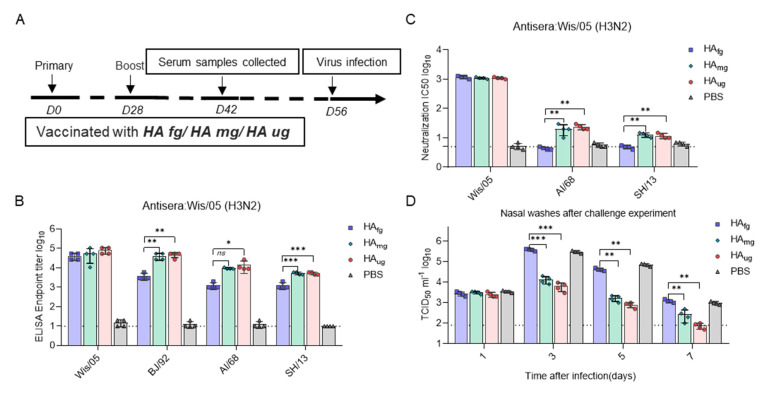
Glycosidase-treated HA vaccines induce protective immunity against influenza virus infection in ferrets. (**A**) Timeline of vaccination and challenge experiments in ferrets. Ferrets (4 ferrets in each group) were immunized with 30 µg of HA_fg_, HA_mg_ and HA_ug_ on day 0 and 28. Sera were collected and measured at 14 days after boost immunization. (**B**) ELISA endpoint titers were determined for Wis/05 (H3N2), AI/68 (H3N2) and SH/13 (H7N9). The immunized ferrets were challenged on day 58 with BJ/92(H3N2). The nasal washes were collected every other day for 7 days beginning at 1 dpi for viral load titration. (**C**) Serum neutralizing antibody titers were determined for Wis/05 (H3N2), Vic/11 (H3N2), BJ/92 (H3N2), AI/68 (H3N2), and SH/13 (H7N9). (**D**) Virus titers were determined by TCID50 assay at 1 dpi, 3 dpi, 5 dpi and 7 dpi post challenge in the nasal washes (NTs) of ferrets. Data are presented as the mean ± SEM. The results were calculated with Prism software using Student’s *t* test; significant differences are marked as * *p* < 0.05; ** *p* < 0.01; *** *p* < 0.001.

The potential of vaccination with a deglycosylated modified HA construct to induce a protective immune response was further evaluated in ferrets, a more sensitive animal model for influenza virus infection [25,26,27]. Ferrets were immunized with doses of 30 μg HA_fg_, HA_mg_, and HA_ug_, and sera were collected on day 14 after boost immunization (Figure 4A). The reactivity of serum collected from vaccinated ferrets against influenza virus was assessed by ELISA and neutralization assays (Figure 4B,C). Vaccination with HA_fg_, HA_mg_, and HA_ug_ all induced broad binding activity to H3N2 and H7N9 viruses, whereas weak neutralization activity was observed in the sera from HA_mg_- and HA_ug_-immunized ferrets, consistent with the immunogenicity results in mice.

In addition, 28 days after the second vaccination, ferrets were challenged intranasally with 50 LD_50_ homologous virus BJ/92 (H3N2). Nasal washes were collected from the infected ferrets. Virus titrations of nasal washes were determined by TCID_50_ assay. Moreover, viral load detection showed that viruses replicated at a lower level in the nasal washes of HA_mg_- and HA_ug_-immunized ferrets at 3 dpi to 7 dpi. At 7 dpi, viruses were not detected in the nasal washes of the HA_ug_ groups, whereas a significantly higher viral titer was maintained in the control ferrets. Thus, these results indicated that HA_ug_-immunized ferrets could provide more effective protection against influenza virus infection.

## 4. Discussion

Despite efforts and the availability of licensed commercial vaccines, seasonal influenza remains a major public health burden worldwide. Antigenic drift, mostly caused by the accumulation of mutations in HA, is the major cause of the mismatch and failure of seasonal influenza vaccines [28,29]. Universal influenza vaccines that elicit protective immunity across all potential subtypes and lineages of influenza viruses would be the holy grail to achieve this goal. The constant addition and removal of glycosylation sites in HA promote viral escape from host immune recognition. It has been shown that glycosylation of HA contributes to protein folding, stability, and biological functions [30]. Furthermore, glycosylation of HA was considered to reduce immunogenicity for immunodominant and neutralizing antibodies by shielding the antigenic sites. As a result, hyperglycosylated HA is designed to mask antigenic sites in the highly variable head domain and concentrate the immune response on the stem domain [18,31]. When a monoglycosylated H1N1 HA-based vaccine (HA_mg_) was reported to elicit a cross-strain immune response, we speculated that deglycosylated modified HA might be beneficial in unlocking occluded and conserved epitopes, especially at the stem region, by removal of surface glycosylation for both globular and stem regions. The major influencing factors, especially for the stem region, contribute to the change in immunodominance patterns. On the other hand, although the prokaryotic expression system does not support glycosylation modification, unstable protein folding might not provide the natural conformation of occluded and conserved epitopes in HA. Thus, rational design for glycosylation modification to unlock occluded and conserved epitopes in eukaryotic expression would be a valuable approach in the future, which would also be applied to live attenuated vaccines to evoke a more robust humoral response to iron out flaws [32,33].

In the present study, deglycosylated modified HA from H3N2 with HA_mg_ and HA_ug_ forms was constructed and evaluated. As a result of increased ADCC and CDC activity and more CD8+ and CD4+ memory T cells, the HA_ug_ vaccine conferred cross-protection against H3N2 and H7N9 subtype viruses. This is consistent with studies showing that ADCC plays an important role in influenza protection [34]. Interestingly, HA_ug_ provides a broader cross-protection ability against the H7N9 virus compared to HA_mg_, which indicated that an Fc-dependent mechanism induced by HA_ug_ might be responsible for protection when there is a lack of in vitro broadly neutralizing activity. The HA_mg_ vaccines were found to be similar or, in most cases, worse than HA_ug_ in cross-strain protection. The reason behind this result is not clear and could be because the amino acid changes from Asn to Asp at each glycosylation site introduced by PNGase F would promote the exposure of protective but occluded epitopes and induce cross-strain protection, while the single N-linker GlcNAc left by Endo-H might cause steric hindrance for those epitopes that are hidden.

As mentioned above, the numbers of glycosylation sites of HA varied following viral evolution and depended on different subtypes and strains. In a previous study, the HA_mg_ vaccines of HA from the A/Brisbane/59/2007 H1N1 strain showed better cross-strain protection against various H1N1 viruses than the HA_ug_ vaccines, in which highly conserved sequences that were originally covered by glycans in fully glycosylated HA are exposed and thus induced more CD8+ and CD4+ memory T cells and IgG-secreting plasma cells. However, the HA_ug_ vaccines from the A/Brisbane/59/2007 H1N1 strain were speculated to contain partial glycans and further influence immunogenicity, which are not completely removed by enzymes. However, the HA_ug_ vaccines from the A/Wisconsin/67/2005 H3N2 strain used in our study contain 11 glycosylated sites and maintain few differences in the secondary structure, while the HA_ug_ from Bri/07 only has eight glycosylated sites. The difference in glycosylated sites and glycosidase treatment may result in the different immunogenicity between HA_ug_ and HA_mg_ vaccines. Although the details to explain this phenomenon are not clear and need to be investigated, the above results could already indicate that deglycosylated modified HA would promote the exposure of protective but occluded epitopes and induce cross-strain protection.

In our results, the low levels of cross-neutralizing activities of the HA_ug_ vaccines would probably not contribute to the cross-protection, especially against A/Shanghai/02/2013 (SH/13). As the predominant antibody present in mice, IgG is the major subtype of HA-specific antibodies with a high affinity for the FcγRIII receptor on immune cells [35]. It was shown that vaccination with the HA_ug_ vaccine increased ADCC and CDC activity along with better protection effectiveness. Indeed, the Fc-dependent functional antibodies would play an important role in unglycosylated HA vaccination, while the modified HA would induce multi-mechanism of protective immune, including more CD4 and CD8 T cell response, even some broadly-protective antibodies with unknown functional mechanisms, which need more exploration and evidence. In summary, our study shows the possibility of deglycosylated modified HA in inducing cross-protection by exposing occluded epitopes and offers insights into design options for more universal influenza vaccines.

## 5. Limitations of the Study

This study focused on deglycosylated modified HA-based vaccine research. The characteristics of the protective epitope changed by PNGase F are still unknown and require further study. In addition, the antibodies with ADCC activities induced by HA_ug_ play an important role in the protective immune response, but how to elicit these antibodies is not exactly understood and requires further study.

## Data Availability

Not applicable.

## Supplementary Materials

The following supporting information can be downloaded at: https://www.mdpi.com/article/10.3390/vaccines10081304/s1, Figure S1: Surface HA glycan was compared and analyzed through the evolution of H3N2 strains; Figure S2: Deglycosylated modified HA elicits a lower IgG1/IgG2a ratio than HA_fg_ against H7 viruses.
