# Supplementary material for: Vaccination with Deglycosylated Modified Hemagglutinin Broadly Protects against Influenza Virus Infection in Mice and Ferrets
Source: Vaccines (Basel). 2022 Aug 11;10(8):1304. doi: 10.3390/vaccines10081304 (PMC9414581; doi:10.3390/vaccines10081304)
Supplement: Supplementary file 1 [file vaccines-10-01304-s001.zip › vaccines-1842942-supplementary.pdf]

A

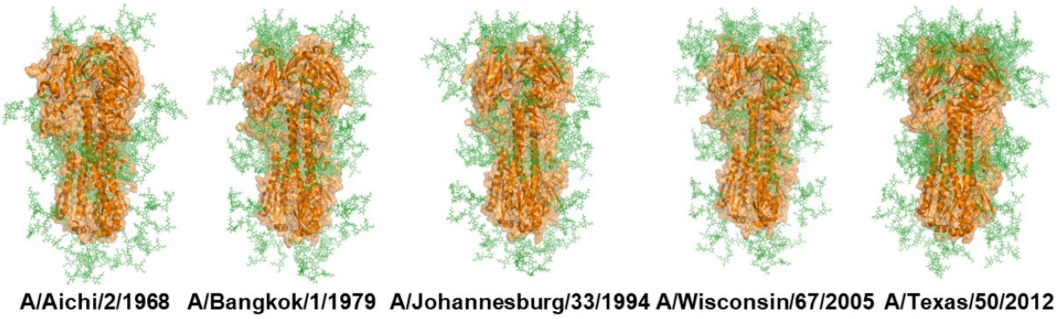

B

Subtype	Virus / H3 numbering	Stem				Head							Stem		Total glycosylation sites
		24	38	54	61	79	138	142	149	160	181	262	301	499	
		NST	NGT	NAT	NSS	NCT	NES	NWT	NGT	NNS	NVT	NST	NGS	NGT	
H3N2	A/Aichi/2/1968	+	+	+		+					+		+	+	7
	A/Bangkok/01/1979	+	+	+		+		+			+	+	+	+	9
	A/Johannesburg/33/1994	+	+	+		+		+			+	+	+	+	10
	A/Wisconsin/67/2005	+	+	+		+		+	+	+	+	+	+	+	11
	A/Kansas/14/2017	+	+	+	+	+	+		+		+	+	+	+	11

C

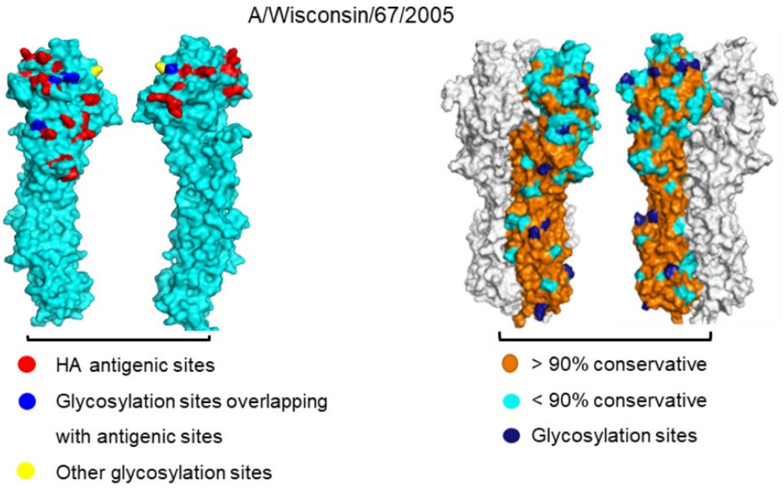

Figure. S1 Surface HA glycan was compared and analyzed through the evolution of H3N2 strains. (A) Structure representation of 1968-2012 H3N2 vaccine strains recommended by WHO with N-glycans attached at their N-glycosylation sites. The protein structure of HAs was created by Discovery Studio and modeled using PyMoL, colored yellow, and the N-linked glycans are displayed in green. All N-glycans were modeled by GlyProt. (B) The details of putative N-linked glycosylation sites for H3N2 vaccine strains. (C) The antigenic epitopes and conserved sites are depicted at the surface of the hemagglutinin protein and overlap with the N-glycosylation sites.

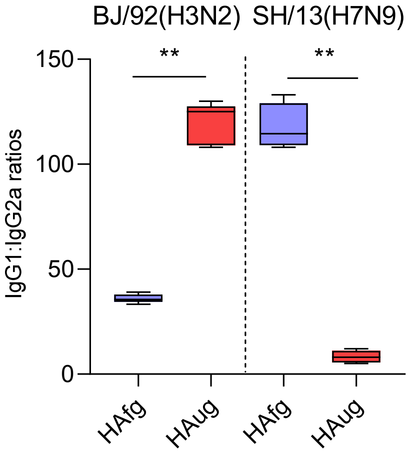

Figure. S2 Deglycosylated modified HA elicits a lower IgG1/IgG2a ratio than HAfg against H7 viruses. The IgG1/IgG2a isotype ratio were measured two weeks after the last vaccination with plates coated with BJ/92 and SH/13. Data are presented as the mean ± SEM. The results were calculated with Prism software using Student's t test; significant differences are marked as *P < 0.05; **P < 0.01; ***P < 0.001.
